# Supplementary material for: Detecting Early-Stage Oral Cancer from Clinically Diagnosed Oral Potentially Malignant Disorders by DNA Methylation Profile
Source: Cancers (Basel). 2022 May 26;14(11):2646. doi: 10.3390/cancers14112646 (PMC9179386; doi:10.3390/cancers14112646)
Supplement: Supplementary file 1 [file cancers-14-02646-s001.zip › cancers-1711428-supplementary.pdf]

Supplementary Table S1. Details of the positive and negative groups in the evaluation of each gene on the training set.

Gene name	Cutoff*		Malignant group	Non-Malignant group
RARβ	1	positive	5	3
		negative	4	28
KLLN	3	positive	5	5
		negative	4	26
CHFR	1	positive	4	1
		negative	5	30
CADM1	1	positive	5	5
		negative	4	26
TP73	2	positive	5	5
		negative	4	26
GSTP1	3	positive	2	1
		negative	7	30
BRCA1	2	positive	3	1
		negative	6	30
ESR1	2	positive	7	12
		negative	2	19
ATM	1	positive	4	5
		negative	5	26
TIMP3	2	positive	4	6
		negative	5	25
BRCA2	3	positive	4	5
		negative	5	26
CASP8	2	positive	4	9
		negative	5	22
MLH	1	positive	5	11
		negative	4	20
APC	1	positive	3	4
		negative	6	27

*The cutoff value indicates the percentage of methylation, calculated by the MS-MLPA method.

Supplementary Figure S1. Methylation profiles are illustrated for the 10 genes with AUC 0.6-0.7 in 9 patients with malignant group and in 31 patients with non-malignant group on the training set. The map shows aberrant methylation (black boxes) or unmethylation (light boxes).

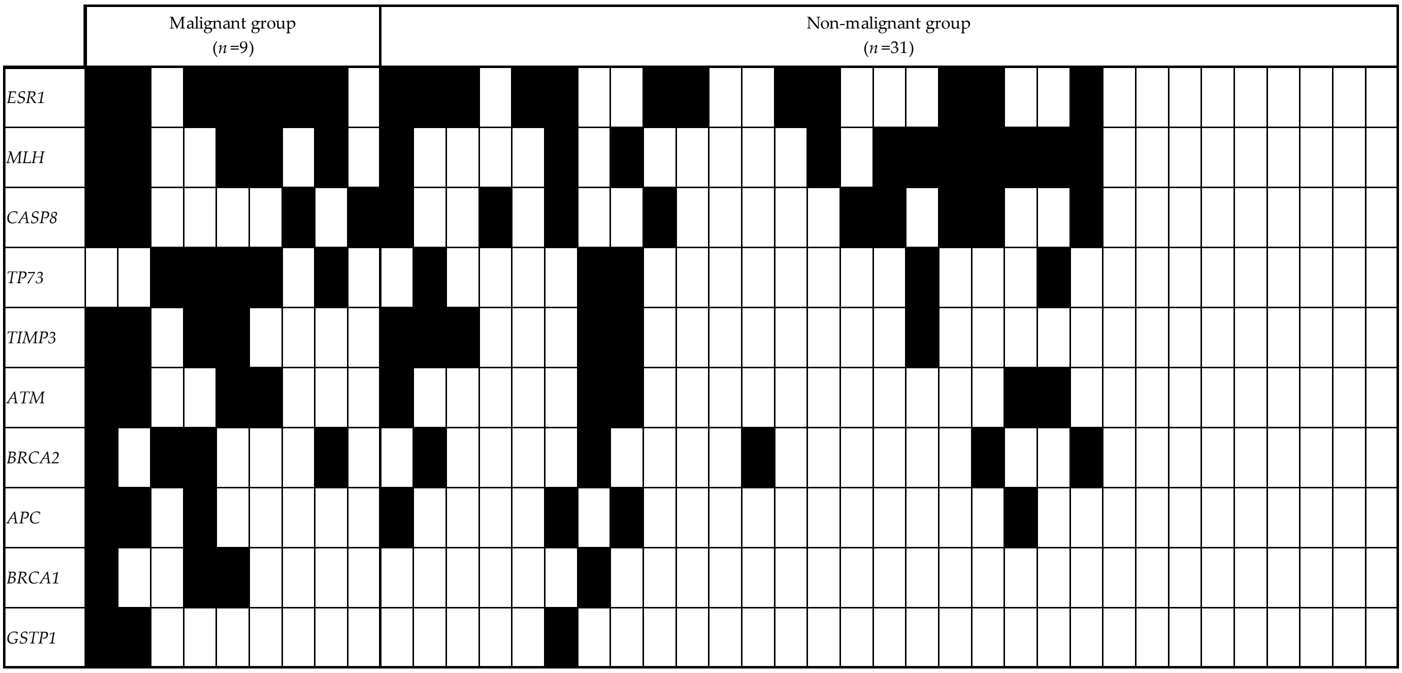

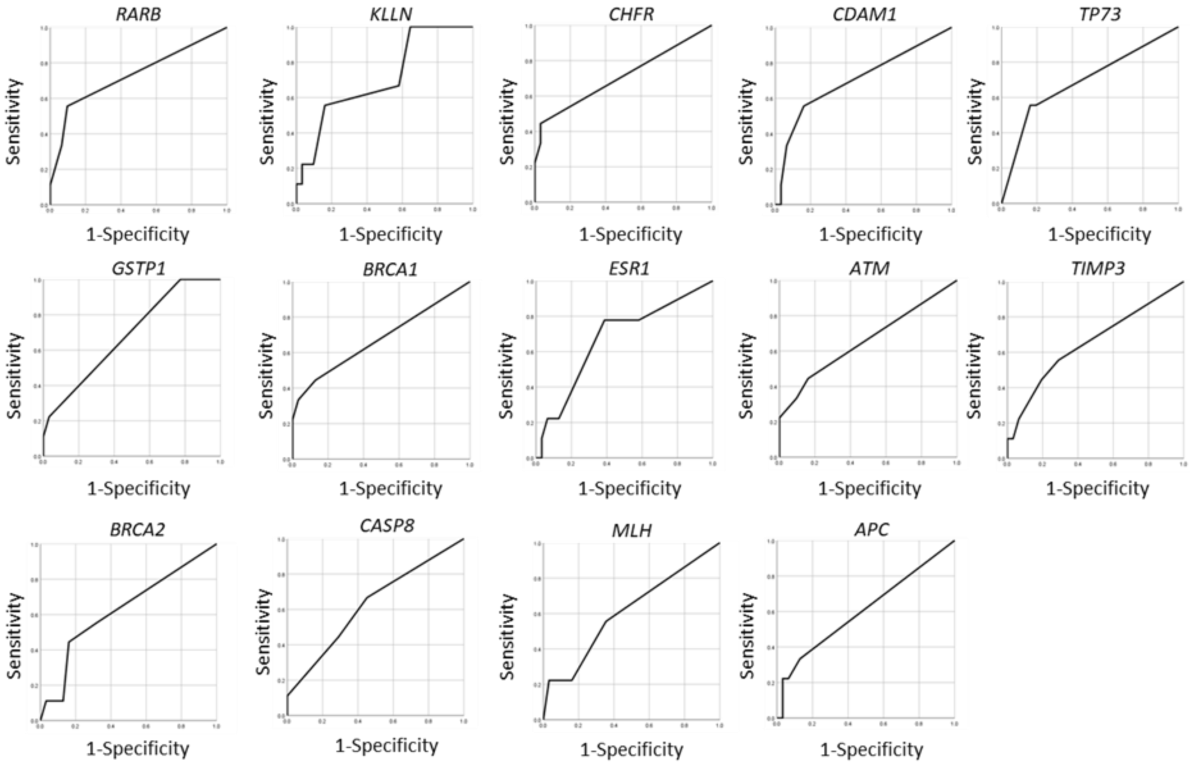

Supplementary Figure S2. ROC analysis for 14 tumor suppressor genes.
